# Pore size engineering of cost-effective all-nanoporous multilayer membranes for propane/propylene separation

**DOI:** 10.1038/s41598-023-48841-8

**Published:** 2023-12-05

**Authors:** Fahime Dehghan, Alimorad Rashidi, Fahime Parvizian, Abdolreza Moghadassi

**Affiliations:** 1 Department of Chemical Engineering, Faculty of Engineering, Arak University, Arak, 38156-8-8349 Iran; 2 Carbon and Nanotechnology Research Center, Research Institute of Petroleum Industry (RIPI), Tehran, P.O. Box 14857-33111, Tehran, Iran

**Keywords:** Chemistry, Energy science and technology, Engineering, Nanoscience and technology

## Abstract

In this study, a new multi-layer hybrid nanocomposite membrane named MFI/GO/ZIF-8 has been synthesized. This membrane combines three nanoporous materials with different morphologies in one membrane without using polymer materials. This allows access to a previously accessible region of very high permeability and selectivity properties. In addition to introducing a new and efficient MFI/GO/ZIF-8 membrane in this work, controlling the pore size of the zeolite layer has been investigated to increase the selectivity and permeability of propylene. The membrane was made using a solvent-free hydrothermal method and a layer-by-layer deposition method. To control the pore size of the MFI layer, a two-step synthesis strategy has been implemented. In the first step, three key parameters, including crystallization time, NaOH concentration and aging time of initial suspension, are controlled. In the second step, the effect of three additional parameters including hydrothermal time, hydrothermal temperature and NH_4_F concentration has been investigated. The results show that the optimal pore size has decreased from 177.8 nm to 120.53 nm (i.e., 32.2%). The MFI/GO/ZIF-8 membrane with fine-tuned crystal size in the zeolite layer was subjected to detailed tests for propylene selectivity and permeability. The structural characteristics of the membrane were also performed using FT-IR, XRD, FESEM and EDS techniques. The results show that the synergistic interaction between the three layers in the nanocomposite membrane significantly improves the selectivity and permeability of propylene. The permeability and selectivity of propylene increased from 50 to 60 GPU and from 136 to 177, respectively, before and after precise crystal size control. MFI/GO/ZIF-8 membrane by controlling the pore size of the zeolite layer shows a significant increase of 23.1% in selectivity and 16.7% in propylene permeability compared to the initial state. Also, due to the precise synthesis method, the absence of solvent and the use of cheap support, the prepared membrane is considered an environmentally friendly and low-cost membrane. This study emphasizes the potential of increasing the selectivity and permeability of propylene in the MFI/GO/ZIF-8 hybrid membrane by controlling the crystal size of the zeolite layer.

## Introduction

Light hydrocarbons are among the most important sources of energy, whose recovery can be used as a source of fuel as well as raw materials for the production of a wide range of chemicals needed by industries. But their separation and purification in the petrochemical industry is very challenging due to their similar physical properties (see Table 1)^1–5^. On the other hand, the environmental and economic problems of common separation methods such as cryogenic distillation, as well as the growth of global demand^6^ for the resulting products, have led researchers to investigate the development of techniques that encourage more efficient separations such as membrane technology.

**Table 1:** Propylene and propane physico-chemical properties^4^.

Gas	Normal boiling point (K)	Molecular weight (g/mol)	Molecular dimension (Å)	Polarizability (10^25^ cm^3^)
Propylene (C_3_H_6_)	225.46	42.08	4.31	62.6
Propane (C_3_H_8_)	231.02	44.10	4.46	62.9–63.7

Conventional polymeric membranes face a fundamental trade-off between permeability and selectivity, known as the 'Robeson upper bound'. To overcome this limitation, several approaches have been developed to construct hybrid or mixed-matrix membranes (MMMs). These membranes incorporate higher-performance nanoporous materials, such as metal–organic frameworks (MOFs) or zeolites, into the polymeric membrane matrix to enhance the membrane performance^7–11^. The incorporation of higher-performance nanoporous materials, such as MOFs or zeolites, into the polymeric membrane matrix can enhance the membrane performance. However, this approach typically results in only a moderate increase in membrane performance compared to pure nanoporous membranes, which have orders-of-magnitude higher performance characteristics than most polymers and polymer-nanoporous material MMMs^10,11^. Despite this, pure nanoporous membranes face a different challenge, namely the difficulty of facile and scalable fabrication of a large variety of membranes for different applications. Unlike polymeric membranes, which can be fabricated in a rational manner with a vast range of polymer compositions, nanoporous membranes require specialized thin film crystallization, growth, or deposition techniques that vary greatly from one material to another^12–20^.

As a result, researchers are interested in using promising membrane materials such as MOFs^21–24^ and zeolites^25–31^, which have been used to make nanoporous membranes without polymer phase one, with the aim of separating hydrocarbons^32–38^. Among zeolite nanostructures and other groups of metal–organic frameworks (MOFs), in the past three decades, the favorable performance of ZIF-8 and MFI nanostructures in gas adsorption and separation as well as the easy synthesis of ZIF-8 and MIF have led to extensive research on the properties^39–41^.

MFI membranes can be grown on various supports. Among these membranes, uniform b-axis membranes with aligned integrated channels can improve separation by reducing the permeation path length, reducing grain boundary defects, and regular arrangement of pores. B-axis MFI membranes are made by different methods. In some cases, TPA^+^ combination can be used as SDA to limit the growth of twin crystals. Xiaofei Lu and his colleagues developed methods such as reducing the concentration of TPAOH, adding ammonium salts, neutral synthesis and ultra diluting synthesis solution for this purpose^42^. Although most of the mentioned methods for the preparation of b-axis membranes have been based on synthetic solutions/gels, Yoon and Tsapatsis' group fabricated a gel-free oriented MFI membrane^43^.

The use of solvents in alkaline synthesis solutions can cause safety and environmental problems. Therefore, it would be very attractive to develop a new solvent-free crystallization method that leads to the fabrication of zeolite membranes with a low density of lattice defects, holes, or cracks. Techniques such as using F- have also been used in this field. Like what Xiaofei Lu and colleagues did. They also developed a highly b-oriented MFI zeolite film by a solvent-free secondary growth method with the help of a synthetic powder containing TPABr and NH_4_F.

The focus of the present article is to further develop the concept of fully nanostructured hybrid membranes through a more detailed study of their synthesis processes and separation properties. The MFI/GO/ZIF-8 system can be an excellent platform for studying fully nanostructured hybrid membranes due to its well-defined coating layers for MFI membranes and the permeability properties of all three materials. Therefore, we have used this system in our work. In particular, we have extended the construction of fully nanostructured hybrid MFI/GO/ZIF-8 membranes using the layer-by-layer deposition technique on a low-cost mullite support. This construction includes 3D MFI and ZIF-8 nanoparticles, as well as 2D graphene oxide nanosheets. In addition, we use Design-Expert software for designing experiments and controlling the pore size in order to produce high-quality MFI layers. This layer is prepared by a two-step hydrothermal method without a solvent, with the seeds being attached to the active support. The advantages of this method include less penetration of MFI layer crystals into the support pores, lower material consumption, absence of a solvent, and the use of a very simple method for coating the support surface and better control of the membrane pore size. As we will show in this article, precise control of the pore size of the MFI layer in the MFI/GO/ZIF-8 nanocomposite membrane can have a significant impact on the permeability and overall selectivity of the membrane.

## Experimental

### Materials

All chemical substances were acquired from industrial companies in reagent grade and used without additional purification. Zn(NO_3_)_2_⋅6H_2_O (Sigma-Aldrich, 99%), 2-methylimidazole (2-MeIM, Sigma-Aldrich, 99%), tetrapropylammonium bromide (TPABr, 98%, Sigma–Aldrich), tetraethyl orthosilicate (TEOS, 99.9%, Merck), silicon dioxide (Sio_2_ ,99.8%, Merck), sodium hydroxide (NaOH, 99.9% Merck), polyethylenimine (PEI, mean Mw ~ 25,000 by LS, Sigma–Aldrich), ammonium fluoride (NH_4_F, Sigma-Aldrich, 99.99%) and Porous mullite supports with a diameter of 2 cm were purchased from Nano Palayesh Seram Company (Iran).

All solvents, such as methanol (MeOH, Merck, 99.9%) and deionized (DI) water were used as received. Propylene and propane (99.99% grade polymer) were supplied by Shazand Petrochemical Company (Arak).

Graphite powder and hydrogen peroxide (H_2_O_2_, 30.0%) were obtained from Sinopharm Chemical Reagent Co. Ltd. (Shanghai, China). Sulfuric acid (H_2_SO_4_, 98.0%), potassium permanganate (KMnO_4_), hydrochloric acid (HCl, 37.0%), and sodium nitrate (NaNO_3_) have been received from XiLong Chemical Co.

### Substrate pretreatment

Before synthesizing the nanoparticles and preparing the membrane, the disk-shaped supports with a diameter of 2 cm were subjected to an ultrasonic acetone bath for 40 min to clean the possible contamination of their surface and pores^44^ . They were then boiled in acetone (56.2 °C) for three minutes and then dried under atmospheric conditions.

### Synthesis of graphene oxide

In this work, the modified Hammers method^45^ was used for the synthesis of graphene oxide. For the synthesis of graphene oxide, it is very important to keep the temperature constant during the reaction stages. In this work, the temperature was controlled below 10 °C at the beginning of the reaction with the help of a thermometer that was placed inside the reaction container. To begin, a glass container was placed in an ice bath and 46 mL of sulfuric acid and 2 g of graphite were poured into the container and stirred completely for 15 min until complete mixing. After that, 6 g of KMnO_4_ powder was slowly added to the previous container while stirring vigorously for 90 min. After finishing the amount of KMnO_4_ powder, the mixing continued for another 30 min. At this stage, the temperature increased to 35 °C and after it became stable, mixing was done for another 60 min. To perform the dilution step, first the stirrer was turned off and 100 mL of distilled water was carefully added to the reaction vessel. Also, another 300 mL of distilled water was poured into a larger container, and the contents of the original container were slowly transferred to the container containing 300 mL of distilled water, and mixing was done again for 30 min, while the temperature at this stage should not be higher than 40 °C. At this stage, the color of the prepared solution changed to light yellow for a short time. To wash the mixture and remove ions and increase the pH, hydrochloric acid and distilled water were added to the mixture and mixed for 60 min. Then, the reaction vessel was placed in a stationary place for 4 h and the settled material was filtered and dried with the help of an oven at 50 °C for 2 h. The dried product is graphene oxide nanosheets.

### Synthesis of MFI zeolite nanoparticles and MFI membrane

MFI nanoparticles were prepared without the use of TPAOH and thru a solvent-free secondary growth method, much like the work of Xiaofei Lu et al.^46^.

Preparation of seed crystals: natural silica MFI zeolite (silicalite-1) seed crystals were fabricated using conventional hydrothermal synthesis. The molar composition of the synthesis solution becomes 1TEOS: 0.3 NAOH: 0.3 TPABr: 100 H_2_O. An obvious homogeneous solution turns out to be obtained through consisting of TEOS drop via drop to a solution of NaOH, TPABr, and H_2_O under full of life stirring for 24 h at room temperature. Then the acquired clean answer changed to be kept at room temperature for 6 h. After that, the solution became transferred to a Teflon autoclave and placed for 20 h at a temperature of 373 K. After crystallization, the autoclave turned being taken out of the oven and positioned at room temperature. Then the resulting crystals are centrifuged and dried in an oven at a temperature of 373 k.

Preparation of Silicalite-1 seed layers for secondary growth: before the usage of the base, they were sonicated in acetone for 1 h. Then they were washed again for 20 min in boiling water and dried in an oven at 373 k for someday. Silicalite-1 b-oriented crystals were covered on the base surface, after which they were rubbed clockwise on the base surface with a finger. To avoid finger infection, latex gloves have been used. A monolayer of b-oriented grains became received after rubbing for numerous mins. To avoid falling off the seed layer, the bases with dense layers of seed were preheated in an oven at 523 K for 3 h before use.

Solvent-free secondary growth: in a standard run for solvent-free secondary growth of b-orientated MFI zeolite films inside the synthetic powder with a molar ratio of 1SiO_2_:0.035TPABr:0.05NH_4_F, 0.50 g of SiO_2_, 0.078 g of TPABr and 0.017 g of NH_4_F have been delivered right into a mortar and combined collectively by way of grinding. After grinding for ~ 8 min, the powder changed into transferred to cover the seeded support in a Teflon-lined stainless steel autoclave (15 ml) for secondary growth at one 175 °C for a sure time (e.g., 2 to 24 h). The typical position of the seeded substrate becomes horizontal at the lowest side of the autoclave and the amount of the artificial powder is turned to ~ 0.58 g (silica = 0.5 g) until precise in any other case.

### Synthesis of ZIF-8 nanoparticles

Similar to the work of Thompson et al.^47^, two solutions have been organized: solution (1) 1.5 g of Zn (NO_3_)_2_.6H_2_O changed into dissolved in 50 ml of methanol. Solution (2) 1.67 g of 2-methylimidazole becomes dissolved in 50 ml of methanol. Solution (2) became brought to solution (1) and it was stirred at room temperature for 1 h with the help of a magnet till a uniform white solution became obtained. Then the obtained white solution was washed in three instances with methanol and centrifuged at 9000 rpm for approximately 5 min each time. The produce becomes dried in an oven at 373 K for one day.

### Preparation of MFI/GO/ZIF-8 hybrid membrane

0.5 g of graphene oxide in 15 ml of water changed into being subjected to an intense stirrer for 4 h, and after the stirrer, it completely dispersed in water with the help of ultrasound. For higher connection of the graphene oxide layer to the layer of MFI membrane crystals, complete dispersion of as much as 4 wt% polyethylenimine in graphene oxide solution in water for 1 h became used. Then, the MFI membrane prepared from the previous steps is immersed in the prepared solution for 10 s to deposit and create the second layer. The prepared bilayer membrane of MFI/GO is then dried in an oven at 433 K for 24 h.

**Figure 1:**
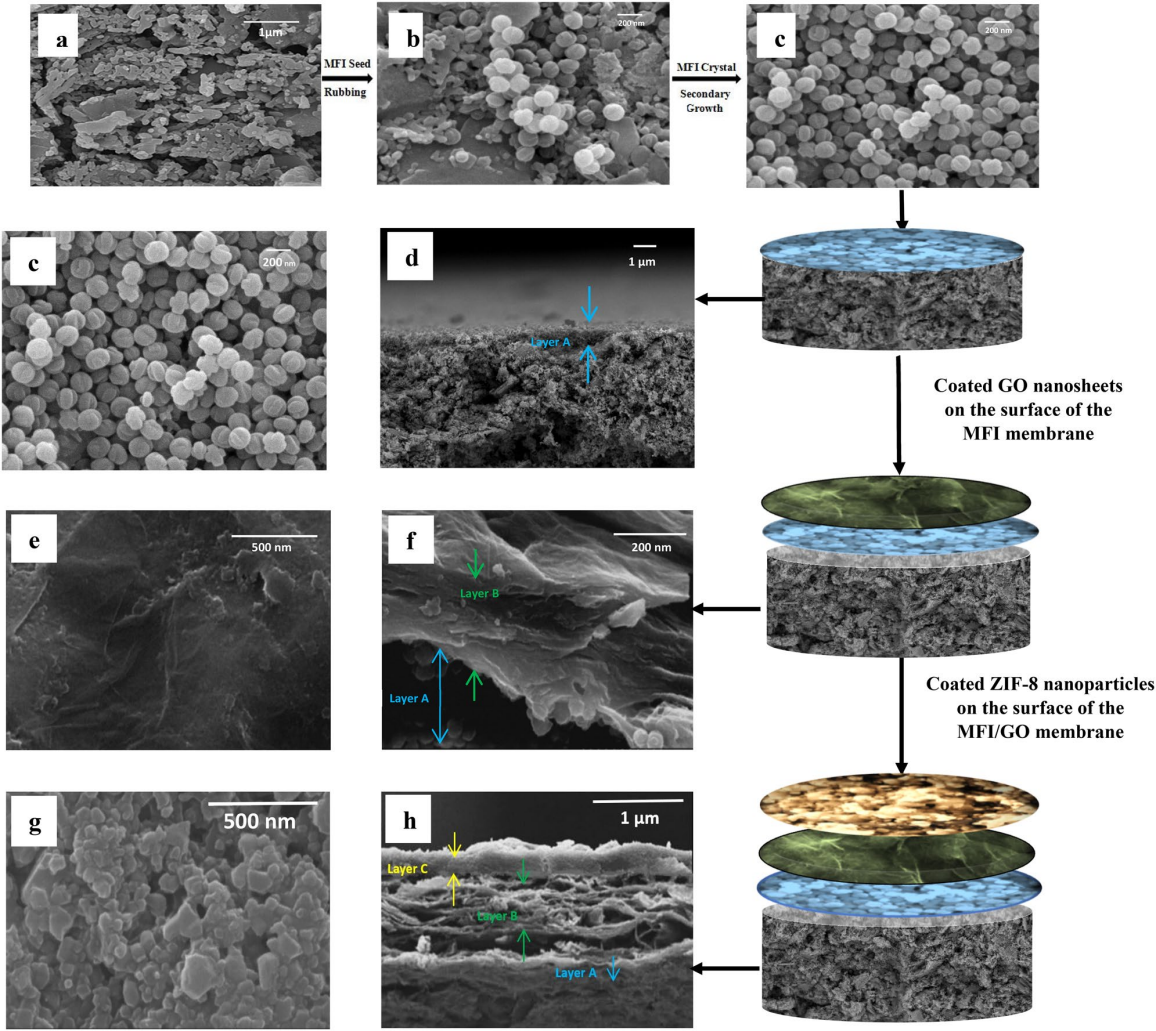
Preparation steps of the MFI/GO/ZIF-8 hybrid nanoporous membrane. FESEM image (**a**) mullite support surface, (**b**) mullite surface after rubbing MFI grains. Surface and cross-sectional images, respectively, (**c,d**) MFI layer coated on the support (formation of MFI membrane), (**e,f**) graphene oxide layer coated on MFI layer (formation of MFI/GO membrane), (**g,h**) Zif-8 layer coated on GO layer (formation of MFI/GO/ZIF-8 membrane).

**Figure 2:**
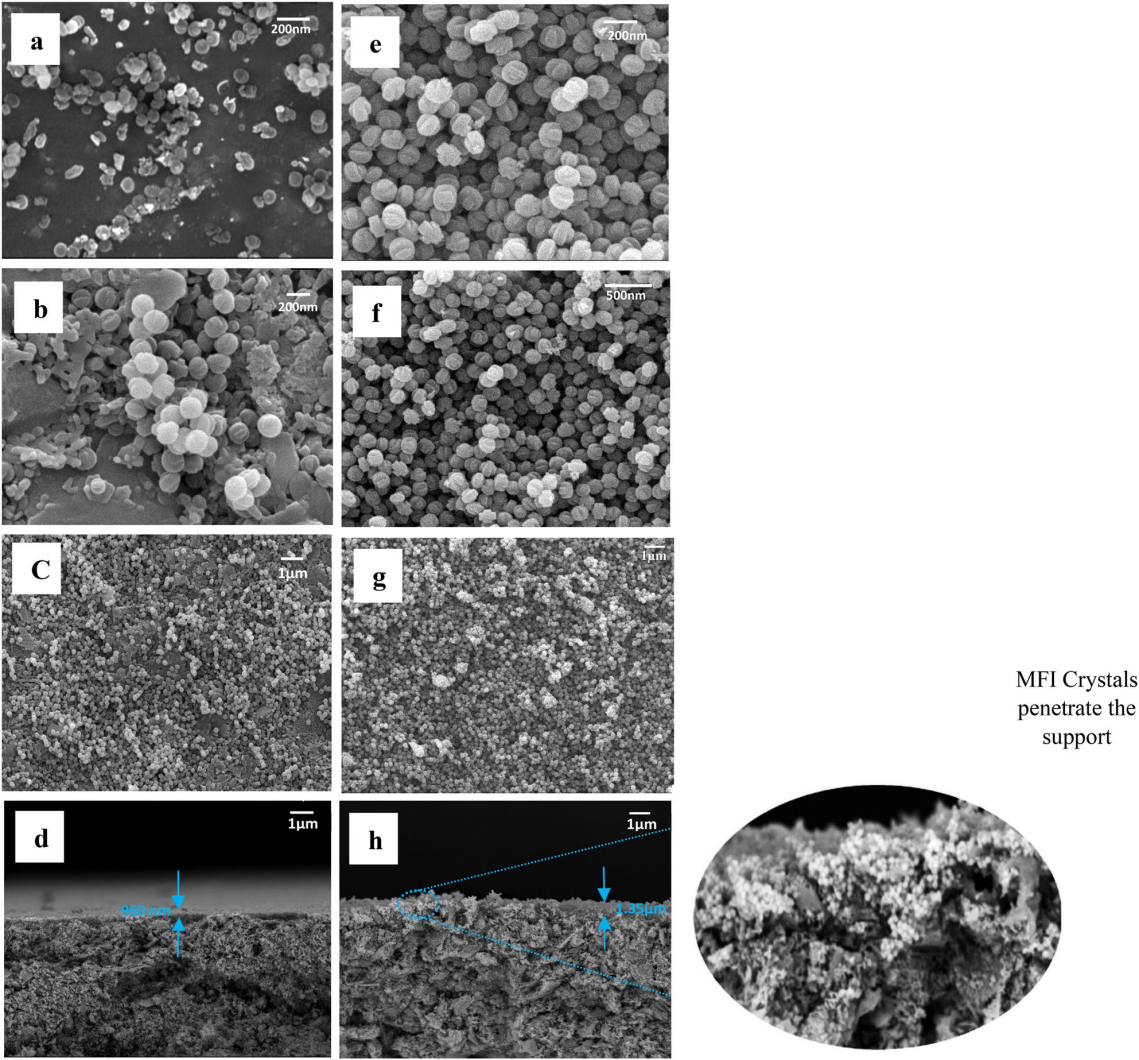
FESEM images with magnifications of 200 and 1 µm (**a–c**) grains deposited by rubbing on the mullite support surface, (**e–g**) secondary growth of MFI crystals on the mullite support surface and Cross-sectional FESEM images (**d**) grains deposited by rubbing on the surface of the mullite support before secondary growth (**h**) growth of MFI crystals on the surface of the mullite support after secondary growth.

A decided number of ZIF-8 nanoparticles prepared from the preceding steps in conjunction with 4 wt% of polyethylenimine changed into dispersed in 15 ml of methanol below extreme and continuous stirring for 4 h. Then, the MFI/GO membrane prepared from the previous steps becames immersed in the prepared solution for 10 s to form the third layer. Then, the organized MFI/GO/ZIF-8 three-layer membrane changed into dried in an oven at a temperature of 433 K for 24 h. In Fig. 1, the preparation steps of the MFI/GO/ZIF-8 hybrid nanoporous membrane can be seen with the details of the surface structure and cross section at each stage of formation. In Fig. 2. FESEM images of the surface and cross-section of the grains deposited by rubbing on the mullite support surface (before secondary growth) and also the secondary growth of MFI crystals on the mullite support surface can be seen.

### Characterization of membrane

The surface morphology and cross-section of the Mullite support and all the membranes were characterized through scanning electron microscopy (SEM, Philips, XL 30) at an accelerating voltage of 20 kV on samples with gold deposition. The thickness records can be obtained from record cross-section images of the base and membrane. The FT-IR spectra for the membrane was received by a Nicolet 4700 FT-IR spectrometer. Powder X-ray diffraction (PXRD) data was recorded on a Bruker AXS-D8 Advance. Blended gas permeation measurements were finished using the Wicke-Kallenbach method at 298 K. He was used as a sweep gas at the permeate aspect, whose stress became blanketed at 2 bar in the course of permeation measurements. A web gas chromatography unit (Agilent 7890A GC) became used to decide the permeate composition.

### Gas permeation setup

**Figure 3:**
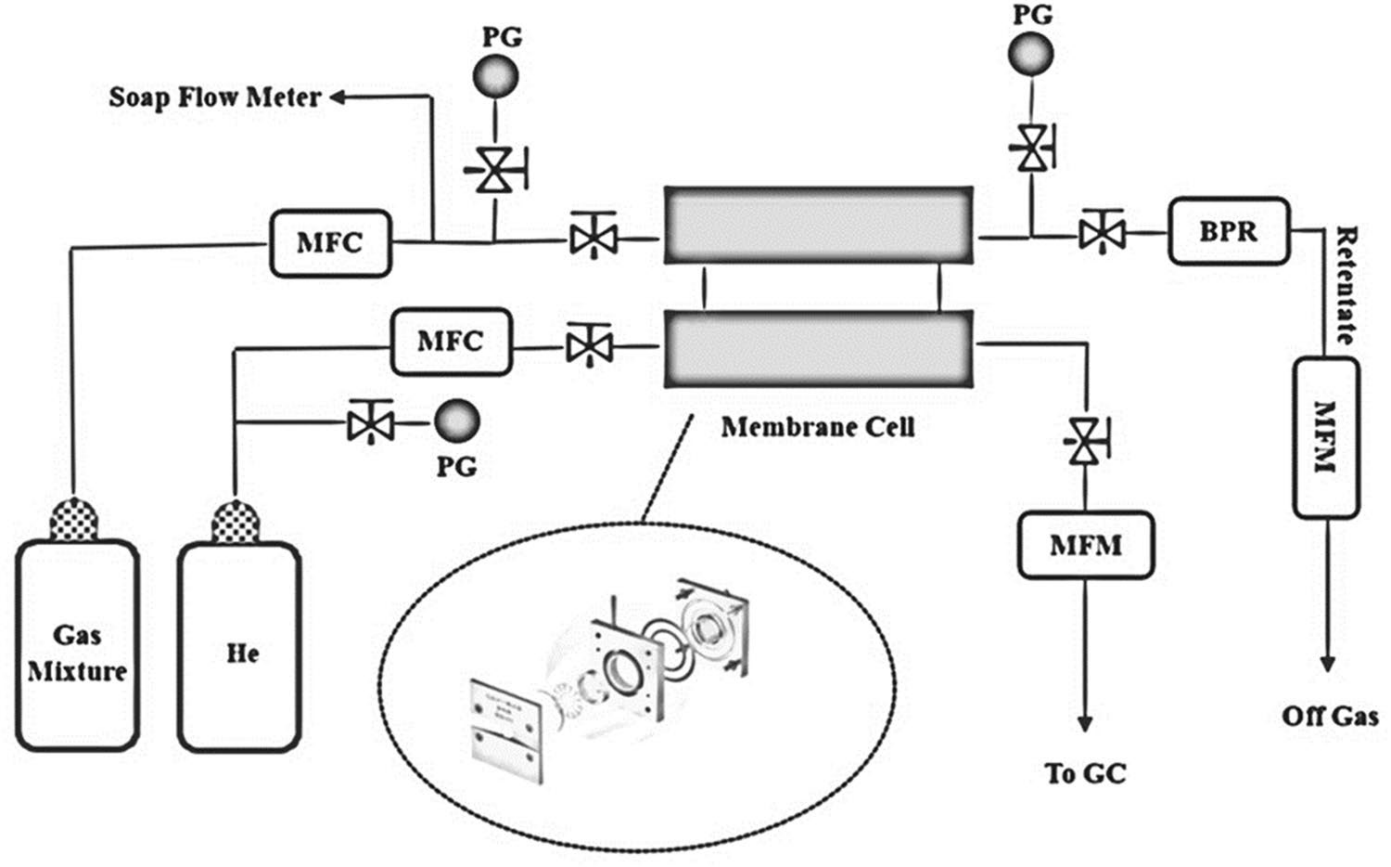
Gas permeation setup.

According to the molecular weight and size of the investigated gases in Table 1, the gas permeability of the constructed MFI/GO/ZIF-8 membrane was measured using a regular strain system (Fig. 3).

For this, a disc-shaped membrane with a diameter of 2 cm, a thickness of 4 mm and an approximate weight of 2.52 g was sealed inside the membrane cell using a Vitron O-ring. Permeate and retentate flow rates for the gas combination C_3_H_6_/C_3_H_8_ = 90/10 were measured by a mass flow meter (MFM), whereas the feed flow rate was controlled by a mass flow controller (MFC). The composition of the feed gas was analyzed by gas chromatography before use. The temperature of the GC column and detector was set at 130 °C, where helium gas was used as the carrier gas at a flow rate of 20 mL/min. A back pressure regulator was used to keep the system pressure constant in the holding line. Percolated and retained flow was measured by digital mass flowmeters. The permeability of C_3_H_6_ and C_3_H_8_ in the MFI/GO/ZIF-8 membrane was investigated at constant temperature and pressure of 298 K and 2 bar. All measurements were performed after 30 min and three repetitions while the gadget had reached its normal condition. Then the average value of permeability and selectivity for the mentioned membrane was presented in Table 2.

**Table 2:** The C_3_H_6_ and C_3_H_8_ permeances and binary C_3_H_6_/C_3_H_8_ mixture selectivity at 298 K with a 90/10 and 50/50 feed mixture at 2 and 1 bar respectively on the MFI/GO/ZIF-8 membrane.

Before pore size control			After pore size control		
C_3_H_6_ permeance (GPU)	C_3_H_8_ permeance (GPU)	C_3_H_6_/C_3_H_8_ Selectivity	C_3_H_6_ permeance (GPU)	C_3_H_8_ permeance (GPU)	C_3_H_6_/C_3_H_8_ Selectivity
50	0.364	136	~ 60	~ 0.34	~ 177

With the help of the observed experimental penetration of the identical molecular species in the multilayer membrane Support/MFI/GO/ZIF-8 (Layer D/Layer A/Layer B/Layer C), much like the work of Kiwon Eum^20^ and with the help of the series-resistance model of penetration in a multilayer membrane as well as the thickness of the determined layers (see Table 2), the inherent permeability of layers A-D can be obtained (see Table 3). Layer A penetrates the porous mullite support, so an extra component ε (as the porosity of the support) need to be considered (here ε = 0.38), which is obtained from mercury porosimetry on the mullite support.

**Table 3:** The thickness of the layers A–D according to the approximate measurement of several SEM images in different places of each layer in the Support/MFI/GO/ZIF-8 membrane and estimated fitted values of C_3_H_6_ and C_3_H_8_ permeabilities and resulting C_3_H_6_/C_3_H_8_ selectivities.

Before pore size control					After pore size control			
Layer	Thickness (μm)	C_3_H_6_ fitted permeability (barrer)	C_3_H_8_ fitted permeability (barrer)	C_3_H_6_/C_3_H_8_ selectivity	Thickness (μm)	C_3_H_6_ fitted permeability (barrer)	C_3_H_8_ fitted permeability (barrer)	C_3_H_6_/C_3_H_8_ selectivity
A	1.560	230.3	0.98	232.7	1.6	328	0.9	364
B	0.588	2706.4	1.36	1981.2	0.49	2193	0.93	2358
C	0.085	18.1	0.208	87	0.07	17.6	0.143	123
D	1.415	433.2	11.3	38	1.94	533.7	9.46	56.3
Total	3.65				4.1			

The permeance of a gas component i in the binary gas system, P_i_ (mol/m^2^ s Pa) was defined by,

1 $${P}_{i}=\frac{{Q}_{i}}{S\times {\Delta h}_{i}}$$

where P_i_ (mol/m^2^ s) is the permeation flux of gas component i in the gas mixture that permeate through the membrane. Δh_i_ is the partial pressure different of component i (Pa) across the membrane, and S is the effective membrane area (m^2^). The binary separation factor for component i (propylene) over component j (propane) is defined as^48^,

2 $${\alpha }_{i,j}=\frac{{P}_{i}}{{P}_{j}}$$

The equation of the infiltration series-resistance model in the three-layer MFI/GO/ZIF-8 membrane, to calculate the intrinsic permeability of the membrane layers, is defined as follows^20^:

3 $$\frac{1}{{P}_{M}}=\frac{{L}_{i,j}}{{p}_{i}}+\dots $$

The experimentally observed thicknesses of a layer of type i found in a membrane of type j are represented by l_i,j_ (with i = Layer A, B, C, D and j = Membrane 1, 2, 3, 4). To derive the intrinsic permeability of layers A-D (P_i_, with i = A, B, C, D), the above equations need to be solved simultaneously, based on the experimental permeability values and the determined thickness of the membranes presented in Table 3.

The overall permeability of the MFI/GO/ZIF-8 membrane can be determined using Eq. (4), assuming the series resistance model^20^:

4 $${\overline{P} }_{overall}=\frac{1}{\frac{{L}_{A}}{{P}_{A}}+\frac{{L}_{B}}{{P}_{B}}+\frac{{L}_{C}}{{P}_{c}}+\frac{{L}_{D}}{{\varepsilon .P}_{D}}}$$

In this equation, L_i_ represents the thickness of the layer (where i = A, B, C, D) in micrometers, and P_i_ indicates the permeability of the layer in Barrer units.

### Box–Behnken methodology

The RSM method uses several experimental designs, such as full factorial, central composite, and Box Behnken. Central Composite (CCD) and Box Behnken (BBD) designs underperform compared to the full factorial design when all linear, interactive, and quadratic effects are considered. In addition to more runs on the same agents, the CCD method has some points outside the scope of safe testing. The BBD method is a set of quadratic designs or close to rotation based on factorial designs at three levels. BBD is often measured as a relatively efficient and ideal alternative to CCD, because CCD requires more testing, more time, and more costs to obtain the model equation^49,50^. BBD has different applications. For example, the absorption process^51,52^, electroanalytical methods^53^, chromatographic methods^54^, analytical spectroscopic method^55^ and membrane process^56^.

In this study, in the preparation of nanoparticles and finally the membrane with MFI quality and to investigate the effect of the parameters, in the first stage of synthesis (synthesis of Silicalite-1 grains), the effect of three parameters: crystallization time, NaOH concentration and aging time of the solution and for the second stage (secondary growth of MFI nanoparticles) the effect of three parameters of hydrothermal temperature, NH_4_F concentration and hydrothermal time in total by running 34 experiments using Behnken box design.

## Results and discussion

**Figure 4:**
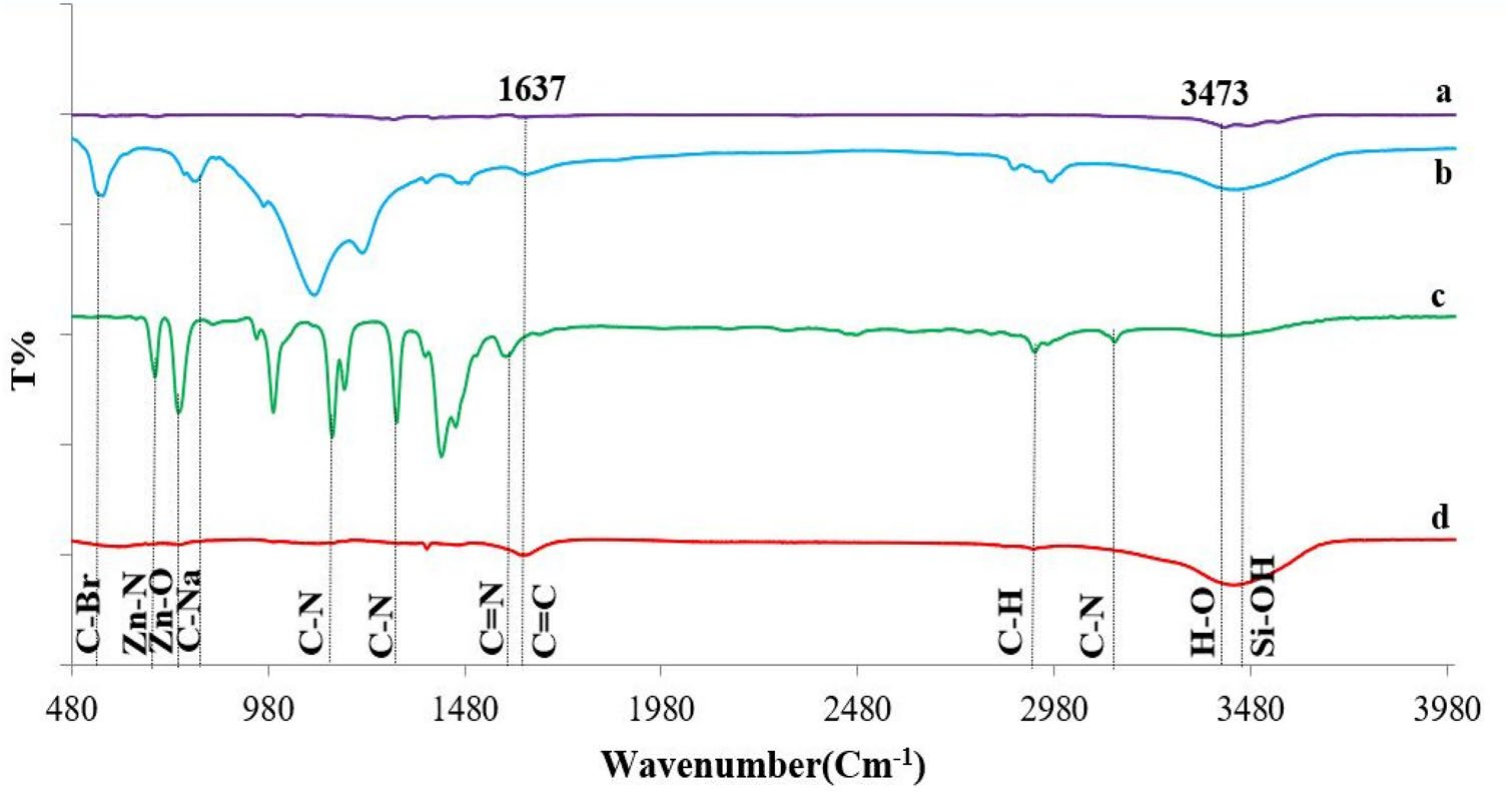
FT-IR results of (**a**) graphene oxide, (**b**) MFI, (**c**) ZIF-8 and (**d**) MFI/GO/ZIF-8 membrane.

The FT-IR spectra of graphene oxide, MFI zeolite, ZIF-8 and MFI/GO/ZIF-8 membrane is presented in Fig. 4. The 3473.45 cm^−1^ and 1637.44 cm^−1^ peaks in Fig. 4a (peak 1637.44 cm^−1^ can also be seen in the membrane spectrum of Fig. 4d) are associated with the H–O stretching vibration at the carboxyl institution and C=C of sp^2^ oxidized carbon bonds in graphene oxide, respectively, which might be regular with preceding guides^57,58^.

From the FT-IR spectrum of the prepared MFI zeolite pattern (Fig. 4b), asymmetric stretching vibrations at 1220.06 and 1098.03 cm^−1^, symmetric stretching vibration at 1794.53 cm^−1^, double ring vibration at 1559 cm^−1^ and T–O bending vibration (T = Al or Si ) observed the structure of zeolite at 457.99 cm^−1^. Within the discovered spectrum of 3443.55 cm^−1^, because of the phenomenon of molecular sieve, MFI crystals are connected to the loose Si–OH businesses of H-bonds^59,60^. Due to the presence of NaOH and TPABr inside the synthesis answer, the 794.53 and 559 cm^−1^ vibrations are attributed to the C–Na and C–Br bonds (which can also be seen in the spectrum of the membrane in Fig. 4d) with the MFI zeolite framework, respectively, which indicates that the metal species within the MFI zeolite channel are completely mixed have had. Figure 4c suggests the stretching vibration of C=N, C–H of –CH3 and C–N of imidazole ZIF-8 rings, which were observed at 1585.27, 2962.5 and 3133.79 cm^−1^, respectively^61^. Additionally, the spectrum of 1137.01 and 1356.84 cm^−1^ is attributed to C–N bonds inside the imidazole institution, the spectrum of 748 cm^−1^ to Zn–O bonds (Fig. 4d) and 675.81 cm^−1^ to Zn–N bonds, which is associated with the structure of ZIF-8^62^. In general, some specific FT-IR peaks in the synthesized materials are reduced or not clearly observed in the spectrum of the membrane (Fig. 4d), which indicates the connection between the functional groups of the nanoparticles forming each layer with other layers and the successful combination of the membrane layers^63–65^.

**Figure 5:**
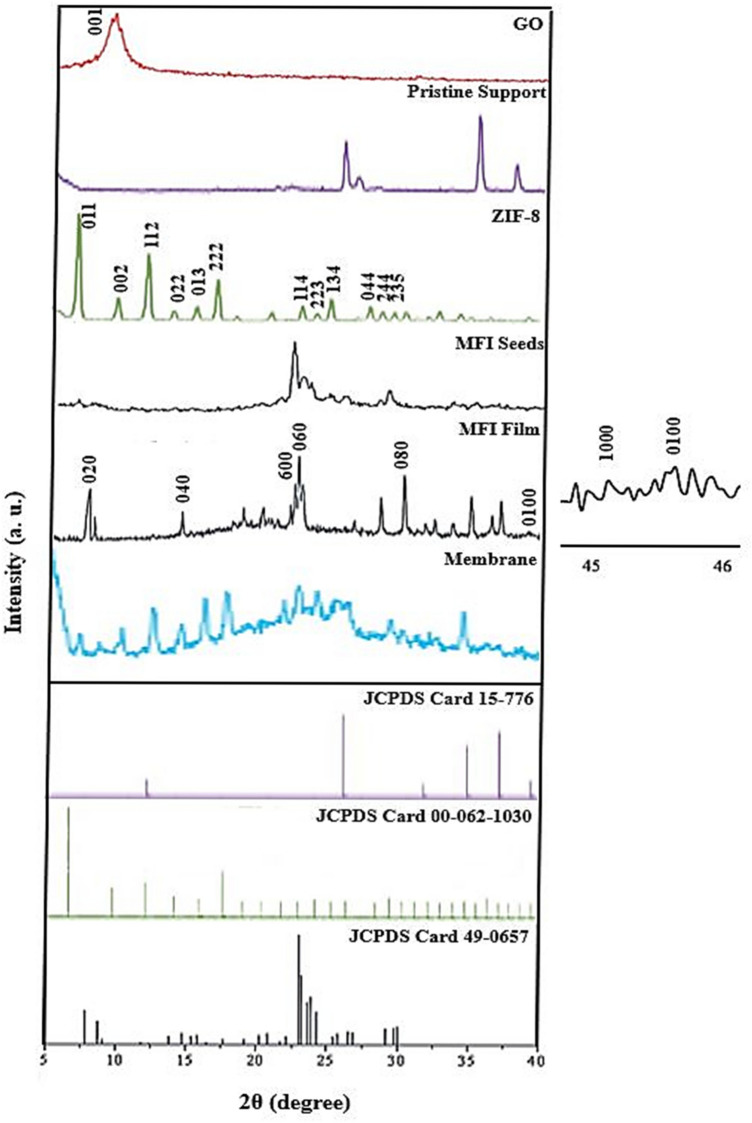
XRD patterns of pristine support, MFI film, MFI grains, ZIF-8, GO and MFI/GO/ZIF-8 membrane.

According to the MFI film XRD patterns of the prepared samples in Fig. 5, the existence of 020 planes at the 2θ = 8° peak, 040 at 16.9°, 060 at 26.5°, 080 at 35.5° and 0100 at 45.5° confirmed the successful growth of the MFI film on the mullite substrate. Also, the wavelengths of 45°–45.5° and 46°, which are attributed to the diffraction of 1000 and 0100 planes, show that the crystallographic direction of the MFI film is the b axis. In addition, the diffraction of 1000 and 0100 planes as a single peak indicates that during the synthesis stages of the MFI film, stacking did not occur and twin crystals were not formed, so a thin continuous film of MFI zeolite was created on the mullite support.

Characteristic peaks at 2θ = 8° and 23.5°, respectively, corresponding to 101 and 105 planes appeared with some other carrier peaks in the MFI grain pattern, indicating the presence of zeolite with pure phase. As can be seen in Fig. 5, the characteristic diffraction peak of exfoliated GO observed in 2θ = 11° can be attributed to the 001 plane cut, which has an interlayer space of 0.9 nm, due to the introduction of oxygen-containing functional groups, water molecules, and other molecules after oxidation related^66,67^.

**Figure 6:**
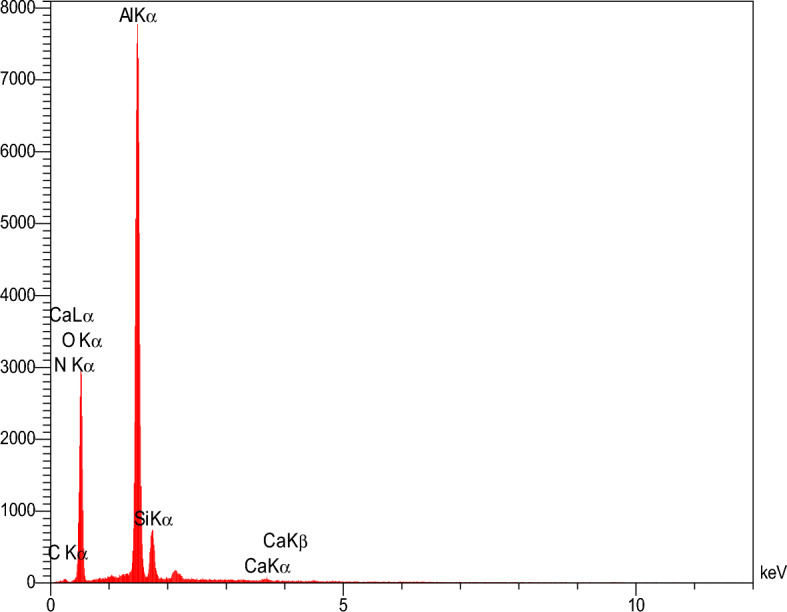
EDS analysis results of Mullite support.

Plates 011, 002, 112, 022, 013, 222,114, 223, 134, 044, 244 and 235 with peaks 2θ = 7.3°, 10.35°, 12.70°, 14.80°, 16.40°, 18°, 24.4°, 26°, 27°, 29.5°, 31° and 33° according to the XRD pattern of nanoparticles ZIF-8 which has been confirmed in previous publications^68,69^. Figure 5, the XRD pattern of the pristine mullite support indicates that the silicate glass segment at 2θ = 20°–30° is identified because of the function diffraction height of the glass phase^70^. Page 110 at 2θ = 15°–20° corresponding to the peak 16.59°, page 012 at 2θ = 25°–30° corresponding to the peak 25.74°, pages 001 and 220 at 2θ = 35°–30° corresponding to the peak 35.34° and pages 002 and 331 at 2θ = 60°–70° are related to the peaks of 57.79° and 68.39° according to the used support compounds (see Table 4 and Fig. 6), which have already been confirmed in the published publications^71–73^.

**Table 4:** Quantitative results of mullite support.

ELT	W%
C	4.37
N	1.97
O	52.10
Al	36.71
Si	4.58
Ca	0.26
Total	100.00

The absence of the 001 diffraction peak in the diffraction pattern of the membrane indicates the formation of completely exfoliated structures in which all individual graphene platelets lose their stacking after being placed on the membrane. Most of the main peaks of ZIF-8 and MFI in the diffraction pattern of the membrane are removed or very weak. This indicates changes in the morphological characteristics of the membrane and occurs due to the difference in the crystallinity of the materials and the composition of the materials in the membrane layers^74–76^.

## Box–Behnken response surface methodology (BBRSM)

The method of designing experiments with the Box Behnken (BBD) With the help of the experimental modeling technique, which is called the response surface method, it is possible to evaluate the relationship between a set of experimental variables and the obtained results, which is one of the best design software and a multivariate statistical method. In this technique, the pore size control process has the following steps:

- Performing statistically designed experiments
- Estimation of coefficients in a mathematical model
- Predicting the answer and checking the adequacy of the model

Randomization ensures that conditions in one run do not depend on conditions in the next run. The BBD method with the help of Design Expert 13.0.5.0 has been used to randomize the implementation, design experiments, investigate the effects of main factors on the construction of MFI quality membrane and find a set of variables that lead to the maximum separation efficiency^56^.

### Controlling the pore size of the MFI zeolite layer in MFI/GO/ZIF-8 membrane to achieve the maximum separation efficiency using BBRSM

Considering that the synthesis of MFI zeolite nanocrystals is a two-step process, for each step, three effective parameters according to the Table 5 were considered for pore size control. Also, 34 test sets based on BBD were proposed to observe the effect of independent factors on the crystal size of the synthesized grains for both steps (See Tables 5 and 6).

**Table 5:** Independent variables and levels of process for BBRSM.

Independent variables	Symbol	Levels of independent variables		
		−1	0	1
Step 1				
Crystalization time (h)	A	20	34	48
NaOH Concentration (g.m^-1^L^-1^)	B	0.1	0.35	0.6
Aging time (h)	C	4	8	12
Step 2				
Hydrothermal time (h)	A	2	13	24
Hydrothermal temperature (˚C)	B	165	172.5	180
NH_4_F concentration (wt%)	C	0.05	0.15	0.25

**Table 6:** Selected parameters and results obtained from the designed experiment.

Run	A	B	C	Step 1						Step 2		
				The average size of MFI zeolite grain (nm)		%Error	A	B	C	The average size of MFI zeolite crystals (nm		%Error
				Actual value	Predicted value					Actual value	Predicted value	
1	48	0.6	8	409.55	409.67	0.03	24	165	0.15	298.36	297.35	0.3
2	34	0.1	4	355.67	355.42	0.07	13	172.5	0.15	210.36	209.55	0.4
3	34	0.6	12	270.86	271.11	0.09	2	172.5	0.05	190.75	188.36	1.2
4	48	0.1	8	622.2	622.58	0.06	13	165	0.25	200.85	200.71	0.07
5	34	0.6	4	410	410	0	13	172.5	0.15	210.56	209.55	0.5
6	34	0.35	8	550	552.60	0.5	13	180	0.05	120.42	120.53	0.09
7	34	0.35	8	558	552.60	1	13	180	0.25	206.97	207.08	0.05
8	20	0.1	8	97.45	97.33	0.1	24	180	0.15	145.53	144.27	0.9
9	34	0.35	8	562	552.60	1.7	2	165	0.15	210.48	213.22	1.3
10	34	0.35	8	545	552.60	1.4	13	172.5	0.15	203.56	209.55	2.8
11	20	0.6	8	318.2	317.82	0.05	13	172.5	0.15	210.56	209.55	0.5
12	48	0.35	12	344.9	344.52	0.1	13	172.5	0.15	213.6	209.55	1.9
13	34	0.1	12	318.1	318.10	0	24	172.5	0.25	198.22	199.58	0.7
14	20	0.35	4	123.7	124.08	0.3	2	180	0.15	180.86	183.35	1.3
15	34	0.35	8	548	552.60	0.8	13	165	0.05	310	309.86	0.04
16	48	0.35	4	574.65	574.53	0.02	2	172.5	0.25	210.6	208.21	1.1
17	20	0.35	12	177.75	177.87	0.07	24	172.5	0.05	240.67	242.03	0.64

According to part 2.4 and the preparation of MFI zeolite grain (silicalite-1) and crystals according to the Table 5, in the synthetic composition 1TEOS: 0.3 NAOH: 0.3 TPABr: 100 H_2_O, changing the concentration of NaOH, crystallization time and aging time of the synthetic solution and after Hydrothermal synthesis by the described method, MFI zeolite grain crystals (silicalite-1) were prepared and the results are presented in the Table 6.

**Figure 7:**
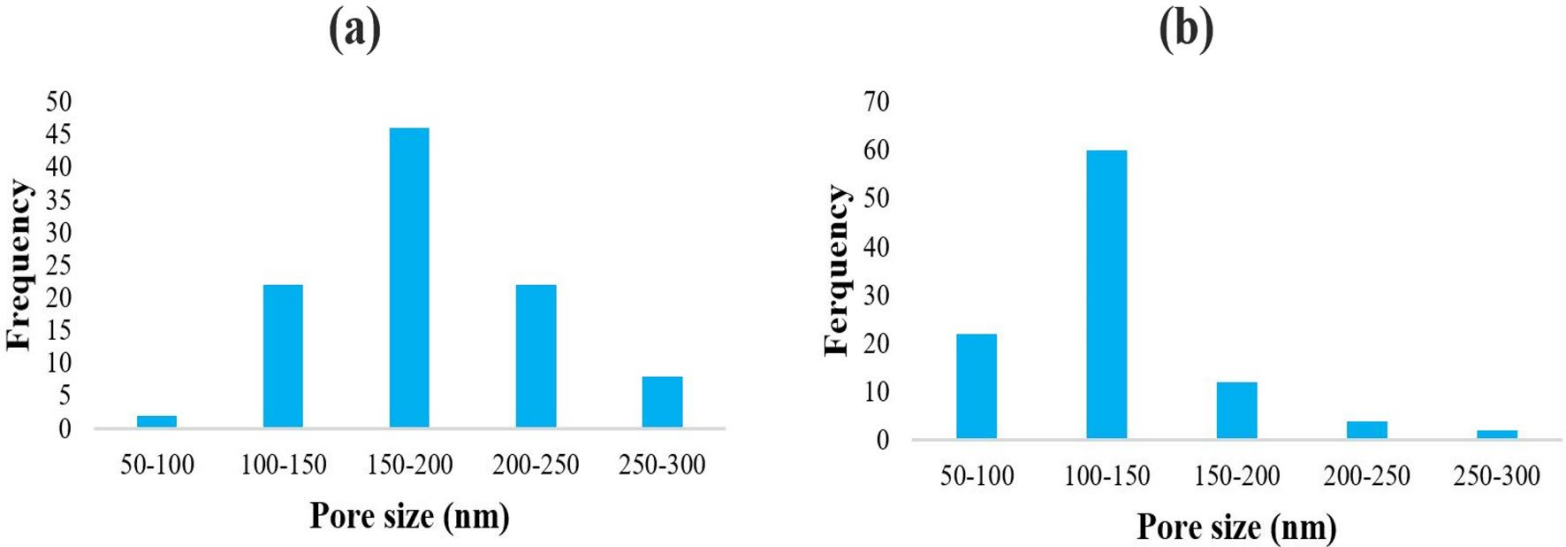
Crystal size distribution (CSD) of 3D MFI nanoparticles, obtained by each randomly analyzing ~ 100 crystals using FESEM. (**a**) Step 1 and (**b**) Step 2.

The results of the investigation on the effect of three selected parameters in the first and second stages of synthesis on the size of Silicalite-1 particles and ultimately the formation of MFI zeolite.

crystals are shown in Table 6. The goal of the experiment design in the first synthesis stage was to find the smallest crystal size of MFI zeolite (Silicalite-1) while considering the maximum frequency distribution, which, according to the results obtained from it, the crystal size formed was controlled in the second stage. Additionally, to ensure the reproducibility of the experiments and prove the normal distribution of the empirical data, the central point parameters were repeated five times in both stages. To select the optimal crystal size, the frequency distribution diagram of Silicalite-1 particles and MFI zeolite crystals (Fig. 7) and their sizes were prepared using ImageJ software, and based on that, the results were presented in Table 6.

### Analysis of variant (ANOVA)

Mean, linear, 2FI, quadratic and cubic models are tested to find the optimal grain size of zeolite MFI (silicalite-1), and the quadratic model and 2FI model are the best models for data analysis in the first and second stages, respectively. One of the most effective parameters in evaluating experimental responses is R^2^. Based on the results reported in Tables 7 and 8, the value of R^2^ obtained for the pore size control of MFI zeolite grain and crystal size (silicalite-1) in the first stage and the second stage are equal to 0.9995 and 0.9973 respectively, which indicates the accuracy of the models^61^. In addition, a significant and very suitable correlation between the self-determining variables and adjusted R^2^ has been suggested. On the other hand, in general, to be meaningful in the model, the difference between predicted R^2^ and adjusted R^2^ should not be more than 0.2. In this work, this difference is 0.0003 in the first stage and 0.003 in the second stage, so the model has high accuracy.

**Table 7:** The ANOVA results of the response surface modified quadratic model.

Step 1	Std. dev	5.40	R^2^	0.9995
	Mean	399.18	Adjusted R^2^	0.9990
	C.V. %	1.35	Predicted R^2^	0.9993
	PRESS	0.7322	Adeq precision	126.8739
Step 2	Std. dev	2.94	R^2^	0.9973
	Mean	209.55	Adjusted R^2^	0.9957
	C.V. %	1.40	Predicted R^2^	0.9931
	PRESS	223.71	Adeq precision	100.4973

**Table 8:** Statistical results of the model for the size response of MFI zeolite grain (silicalite-1) and crystals.

Source	Sequential p-value	Adjusted R^2^	Predicted R^2^
Step 1			
Linear	0.0409	0.3337	0.1200
2FI	0.3163	0.3821	0.0684
Quadratic	< 0.0001	0.9990	0.9993
Cubic	0.9994	0.9982	
Step 2			
Linear	0.0123	0.4525	0.0705
2FI	< 0.0001	0.9957	0.9931
Quadratic	0.9601	0.9942	0.9834
Cubic	0.6031	0.9933	

The F-value indicates the strength of interaction between each independent variable, and the p-value represents the significance of each coefficient. For the model parameters to be significant, the F-value should be greater than one and the p-value should be less than 0.05^77^. The ANOVA results for the first and second stages of controlling the crystal size of MFI zeolite (silicalite-1) are presented in Table 9. As shown in Table 9, the proposed models for studying the process of controlling the crystal size of MFI zeolite (silicalite-1) were significant due to the low p-value (for example, < 0.0001). Additionally, the large significant F-value confirms the model's significance, and with the least error, experimental systems can be modeled. The lack of fit for the models for the first and second stages of the MFI zeolite grain (silicalite-1) and crystal size were equal to 0.9994 and 0.8561 respectively. These values show that the disproportion to the net error was not significant. The Table 7 shows the effective value of each factor, standard errors, standard effect values and regression coefficients.

**Table 9:** Analysis of variance for the modified quadratic and 2FI.

Source	Sum of squares	df	Mean square	F-value	p-value	
Step 1						
Model	4.488E+05	9	49,870.96	1711.64	< 0.0001	Significant
A–A	1.904E+05	1	1.904E+05	6534.99	< 0.0001	
B–B	28.84	1	28.84	0.9899	0.3529	
C–C	15,524.10	1	15,524.10	532.81	< 0.0001	
AB	46,958.89	1	46,958.89	1611.69	< 0.0001	
AC	20,135.61	1	20,135.61	691.08	< 0.0001	
BC	2579.12	1	2579.12	88.52	< 0.0001	
A^2^	52,891.14	1	52,891.14	1815.29	< 0.0001	
B^2^	26,059.64	1	26,059.64	894.40	< 0.0001	
C^2^	77,045.52	1	77,045.52	2644.30	< 0.0001	
Residual	203.96	7	29.14			
Lack of fit	0.7550	3	0.2517	0.0050	0.9994	Not significant
Pure error	203.20	4	50.80			
Cor total	4.490E+05	16				
Step 2						
Model	32,346.29	6	5391.05	625.47	< 0.0001	Significant
A–A	1014.53	1	1014.53	117.70	< 0.0001	
B–B	16,736.27	1	16,736.27	1941.73	< 0.0001	
AB	3795.18	1	3795.18	440.31	< 0.0001	
AC	970.32	1	970.32	112.58	< 0.0001	
BC	9574.62	1	9574.62	1110.84	< 0.0001	
Residual	86.19	10	8.62			
Lack of fit	31.68	6	5.28	0.3874	0.8561	Not significant
Pure error	54.51	4	13.63			
Cor total	32,432.49	16				

### Diagnostic model

**Figure 8:**
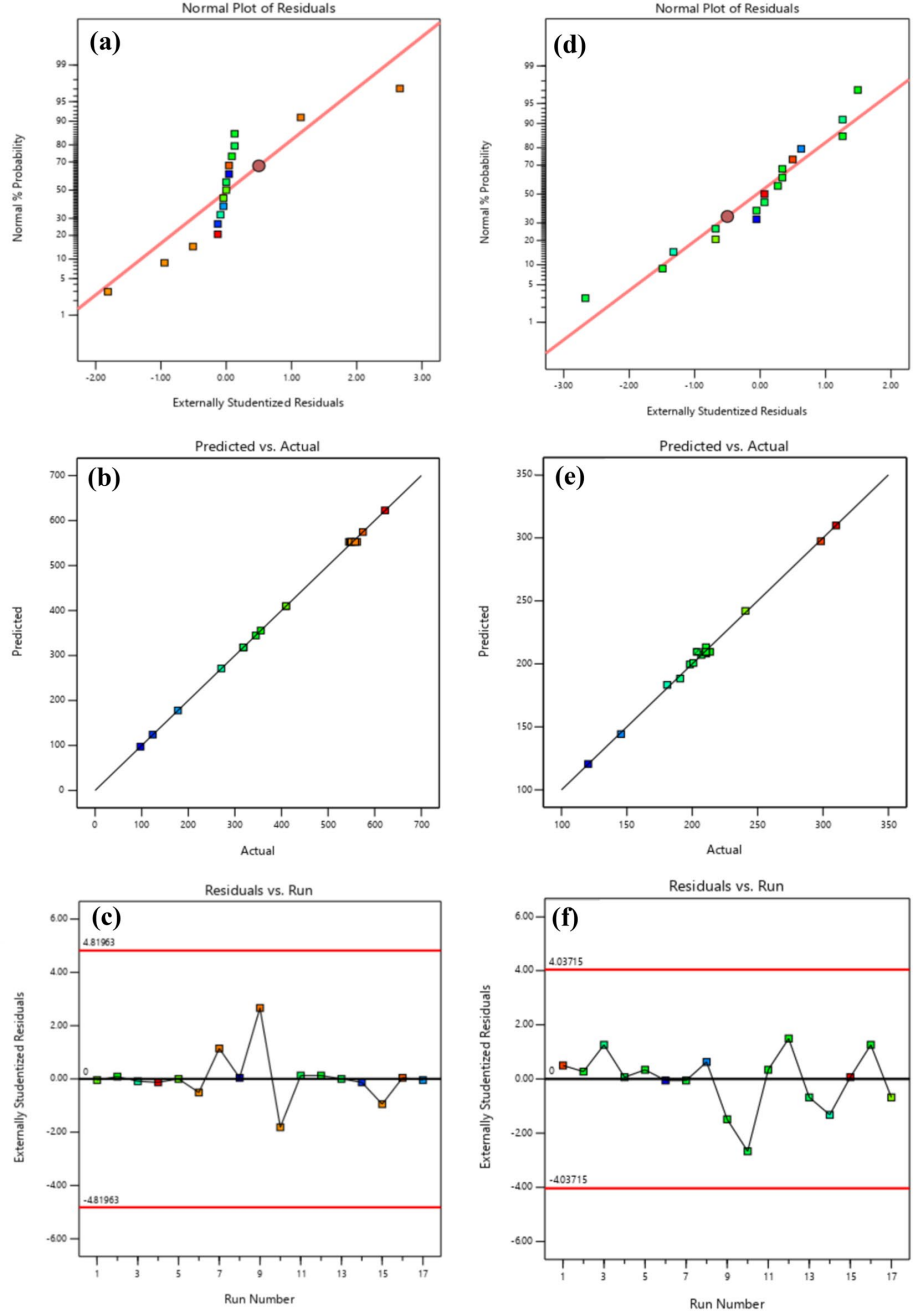
The curve of the normal probability: (**a**) MFI zeolite grain (silicalite-1), (**d**) MFI zeolite crystals, and the curve of the predicted response versus actual response: (**b**) MFI zeolite grain (silicalite-1), (**e**) MFI zeolite crystals; analysis of residual for the response to the (**c**) MFI zeolite grain (silicalite-1), (**f**) MFI zeolite crystals.

The correctness of the data of the proposed model can be examined from another direction, and that is, to examine the normal distribution of the data. According to Fig. 8a,d for the first and second stages of MFI nanocrystals, as it is known, the normal values and actual statistics of the proposed model are close to the straight line, which indicates the normal distribution of the proposed model. Also, Fig. 8b, e confirms the results of experimental data and mathematical model to create the optimal size of MFI zeolite nanocrystals. Residuals analysis is an important indicator that is considered to diagnose the proposed model and predict the response. These plots show how well the model fits the real data. In these graphs, if the points are randomly distributed without any particular pattern, this indicates that the model is consistent with the real data(Fig. 8c,f)^61^.

### Response surface analysis

**Figure 9:**
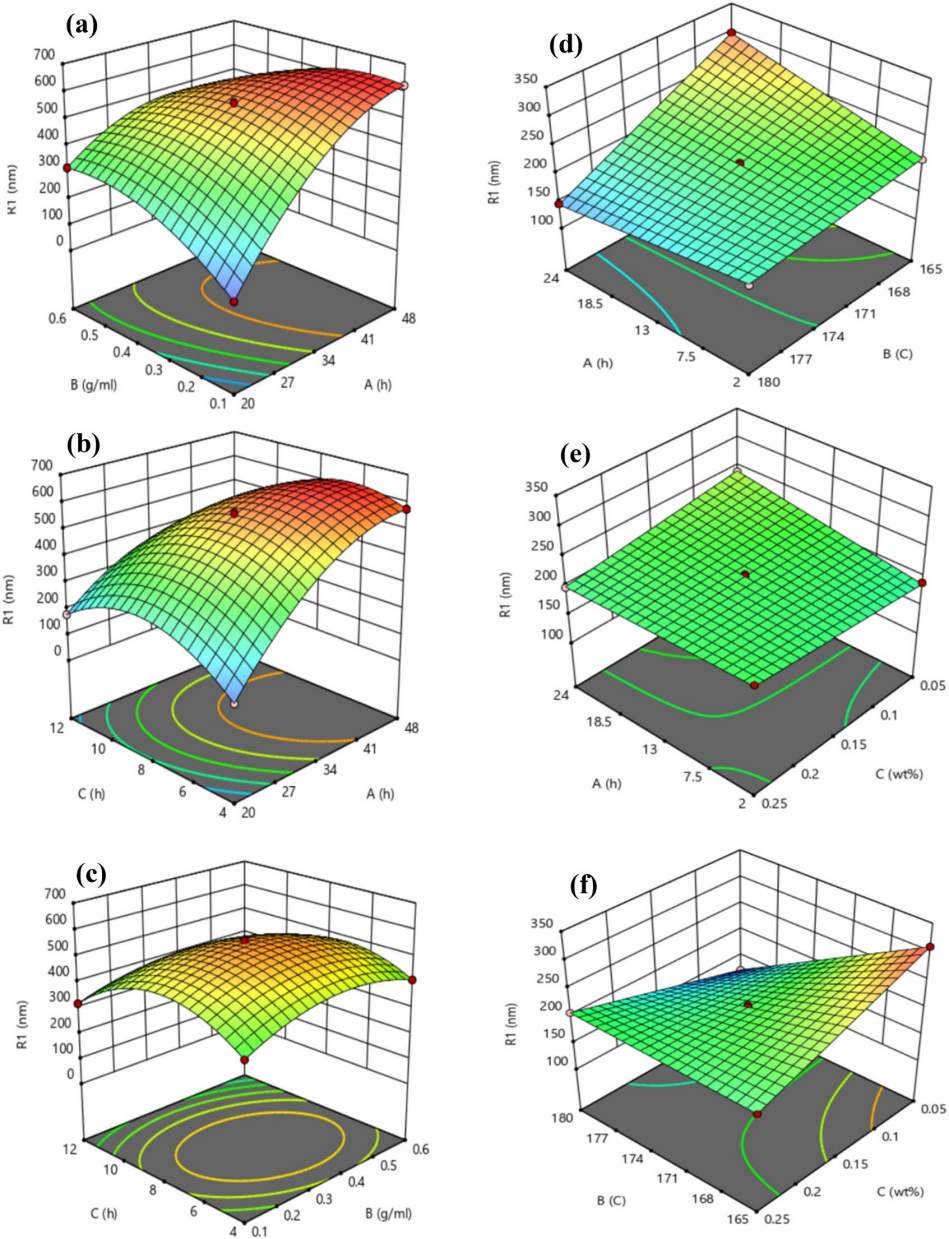
Response surface graphs of MFI zeolite crystal size (**a**) effect of crystallization time and NAOH concentration, (**b**) effect of aging time and crystallization time, (**c**) effect of aging time and NAOH concentration, (**d**) effect of hydrothermal time and hydrothermal temperature, (**e**) effect of hydrothermal time and NH_4_F concentration, (**f**) effect of hydrothermal temperature and NH_4_F concentration.

In the first stage of synthesis, to achieve the optimal size of MFI nanocrystals, three parameters of crystallization time, NaOH concentration and aging time were considered, and in the second stage of synthesis, three parameters of hydrothermal time, hydrothermal temperature and NH_4_F concentration were considered. Graphical display of response surface analysis (3D) was used to study the relationship between the mentioned variables in both stages and to detect the optimal level of each variable to control the nanocrystal size. The 3D quadratic plot of the variables is reported in Fig. 9.

### Analysis of response surface to crystal size of zeolite MFI

A three-dimensional graphical representation was used to study the relationship between aging time of synthetic solution, crystallization time, and NaOH concentration in the first synthesis stage, as well as hydrothermal time, hydrothermal temperature for crystallization, and NH_4_F concentration in the second stage to determine the optimal level of each variable to reduce the size of MFI zeolite crystals. The results are shown in Fig. 9.

Figure 9a illustrates the combined effect of NaOH concentration and crystallization time of the initial suspension in the first synthesis stage to create desired MFI nanocrystals. The effect of crystallization time in MFI zeolite synthesis is crucial due to its impact on the growth rate of crystals, as well as the type and size of the structural zeolite particles. If the crystallization time of MFI zeolites is long, the likelihood of other phases forming in addition to MFI zeolites increases, which can lead to defects in the final zeolite properties. Conversely, if the crystallization time is short, the crystal size will be smaller^78–80^. If the concentration of NaOH directing agents is high, the amount of aluminum ion exchange in the zeolite structure increases, and the crystal size will be larger with a smaller particle size distribution.

Therefore, as shown in Fig. 9a, increasing the crystallization time and NaOH concentration increases the size of MFI crystals (up to about 630 nm), while the effect of increasing NaOH concentration is less than the effect of increasing crystallization time^81–83^. Furthermore, the use of NH_4_F as a crystallization director in the second stage, by creating fluoride ions in the environment, can act as a speed control agent for crystal formation. Therefore, with an increase in NH_4_F concentration, the rate and size of crystal formation increases. However, excessive NH_4_F concentration can also lead to the formation of irregular and nonuniform crystals. Moreover, the rate and size of crystal formation increase with an increase in hydrothermal time (Fig. 9e)^84–86^.

In the second synthesis stage, in addition to crystallization time, the effect of hydrothermal temperature was considered. As shown in Fig. 9d, high temperature can increase the rate of crystal formation. Moreover, at higher temperatures, there is more thermal energy in the system, which can lead to the formation of larger crystals at a faster rate. However, very high temperatures can lead to the formation of irregular and side-phase crystals, and the likelihood of forming crystals with irregular structures increases. In this case, two parameters are almost equally effective on the crystal size, and the maximum size of the obtained crystals is 300 nm^87,88^.

Based on Fig. 9b, with an increase in crystallization time, the size of crystals increases, as there is more time for the connections and formation of crystals at the synthesis temperature, leading to an improvement in the connections, making the crystal particles closer to each other, and simultaneously increasing the crystal size^89,90^. On the other hand, with an increase in aging time, crystals become more open to the chemical compounds present in the environment. This improves the connections between crystal particles and brings them closer to each other. As a result, the overall free energy decreases. Since the crystal formation process can generally continue with a decrease in free energy, crystals become smaller. As visible from Fig. 9b, an increase in crystallization time will result in a much greater increase in grain size than aging time^91,92^. Therefore, a relatively short crystallization time and long aging time can be used to obtain MFI zeolite nanocrystals.

The combined effect of NaOH concentration and aging time on the formation of MFI crystals is shown in Fig. 9c, where an increase in NaOH concentration leads to an increase in the solution's pH, which can help increase the rate of crystal formation. However, excessive NaOH concentration can lead to the formation of a different type of crystal. Moreover, the crystal size increases with an increase in aging time. Additionally, aging time can be accompanied by a delay in crystal formation^93–96^. As a result, to control the conditions of crystal formation, it is possible to help the formation of MFI crystals with a more suitable size by changing the NaOH concentration and aging time. With an increase in hydrothermal temperature, the crystallization time of crystals decreases, leading to an increase in crystal size. This is because at higher temperatures, the rate of crystal crystallization increases, and the crystallization time becomes shorter. Therefore, with an increase in hydrothermal temperature, the crystal size increases. Simultaneously, with an increase in both hydrothermal temperature and NH_4_F concentration, the likelihood of forming larger crystals increases, as seen in Fig. 9f^88,89^. From the influence of the mentioned parameters in the first and second stage, it can be concluded that the most important factor in the first stage was the crystallization time and, in the second stage, the NH_4_F concentration to form the desired size of the crystals.

### Pore size control and validation

In this research, in order to control the selected variables in the first stage, the smallest crystal size of the MFI zeolite grain (silicalite-1) was considered as the target due to their highest abundance, and in the second stage, the crystal size was considered the parameters affecting it were controlled after adjusting the model for fit. The software predicted the optimal values of crystal size to achieve the mentioned goal in the first stage of synthesis of 177.8 nm in 12 aging times, crystallization time of 20 h and 0.35 NaOH concentration, and in the second stage of synthesis 120.53 nm crystal size, hydrothermal time of 13 h, hydrothermal temperature of 180 ˚C and NH4F concentration of 0.05. Laboratory results and predicted results are presented in Table 6.

### Separation properties of the layers

B-oriented MFI films were fabricated on a porous mullite base by careful selection of experimental conditions and precursor compositions. These films, as can be seen in the FESEM images (Fig. 2), do not have twin nuclei and were prepared with the help of secondary growth without using TPAOH as a structure directing agent (SDA), which is more economical than b-Oriented MFI films made in the presence of TPAOH. In this work, TPAOH + NaOH combination was used in both stages of solvent-free synthesis, which led to the creation of a continuous and crack-free film (Fig. 2g) with identical crystals and no bonded or twin nuclei. The size of the MFI grains in the first stage of synthesis was on average 100 nm with a thickness of 960 nm, and after the secondary growth stage, the crystals became larger and merged, and the size of the created MFI crystals was approximately 150 nm and the thickness of the created film on the porous mullite support was approximately 1.35 µm (Fig. 2d, h). The very small increase in the thickness of the film formed in the secondary growth stage compared to the first stage (16%) means that the undesirable growth of the twin nuclei of the MFI grains was inhibited^97^. Even before the secondary growth stage, twin seeds were not observed in our system (Fig. 2c). In addition, the cross-sectional images clearly show dense grain and crystal growth.

From the FESEM images, it can be clearly seen that the growth and nucleation of MFI crystals occurred in the secondary growth stage and only by rubbing the seeds on the support in the previous stage. In Fig. 2, the FESEM images of the seed planting stage by rubbing on the porous mullite support and also the secondary growth stage of the crystals show that silicalite-1 grains cover 100% of the support surface and are fully grown on it^98^. Overall, in this work, we have created an easy and economical path to grow highly b-oriented MFI skinny films on mullite substrate.

The separation properties of a C_3_H_6_/C_3_H_8_ = 90/10 mixture were measured using an MFI/GO/ZIF-8 membrane at a temperature of 298 K and a pressure of 2 bar. The obtained permeation and selectivity values for the mixture are presented in Table 3. In the first layer (pure MFI), a high propylene gas permeation rate of 230.3 Barrer was observed due to the low stability of transport caused by the large pore size of the zeolite channels. Also, a relatively high selectivity (232.7) was obtained in this membrane, which is probably due to the adsorption of propylene molecules in the zeolite membrane by the dual-functional bonds in propylene that can interact with hydroxyl groups of silicon and cations in the zeolite framework^99–101^. On the other hand, MFI zeolite has three-dimensional pores, including two types of intersecting, helical and straight pores, with an average pore diameter of about 0.55 nm. The permeation through b-oriented MFI pores is significantly faster than permeation through a- and c-oriented MFI pores. Therefore, since the pores act directly along the membrane thickness (the prepared membrane thickness is low and about 1.560 µm, b-oriented MFI may provide a faster transfer of molecules, which mainly provide a large volume^102^. After several rounds of coating using the dip-coating method on the MFI layer, the GO layer was deposited. The membrane surface was covered with a very thin layer of GO without any detectable holes or cracks, which had a thickness of 0.588 µm. In this method, polyethylenimine was used as a connector to create a stronger adhesive force between GO nanosheets and the surface of the membrane covered by MFI, as well as to control the size of the surface pores formed at the nanoscale. Individual GO nanosheets are strongly held together by hydrogen bonds and van der Waals forces^103^.

In general, in an alkaline aqueous solution, a number of carboxylic acid groups located in the outer wall cavities are cleaved on the edges of graphene oxide (GO) because of the presence of free carboxylic acid groups. As a result, the edges of the GO sheet become negatively charged due to the ionized pendant carboxylic acid groups. Consequently, the edge-to-edge interactions of GO sheets are repulsive, leading to the formation of island-like GO sheets on the membrane surface. Nanoholes are produced via the edges of the GO sheets, which are shaped with the GO structure, and gas permeation occurs among those nanoholes. Therefore, C_3_H_6_ can act as a Lewis acid or a Lewis base and can participate in hydrogen bonding. Carboxylic acid groups provide a preferred site for C_3_H_6_ adsorption, and as a result, by strongly trapping C_3_H_6_ molecules, they are preferentially adsorbed on the pores of the walls, facilitating their penetration and transfer. On the other hand, propylene is a polar gas, so the polarity of each C-O bond in the propylene molecule allows it to interact with polar groups in GO. Therefore, the use of 2D graphene oxide nanosheets with out-of-plane orientated channels results in the highest increase in permeability, which is determined in layer B (Table 3).

The increase in gas selectivity (2706.4 compared to 232.7 according to the results presented in Table 3) can be attributed to the uniform alignment of graphene oxide nanosheets when placed on the first layer, which minimizes the formation of wrinkles and creates a smoother layer. This high propylene/propane selectivity suggests a molecular sieving mechanism wherein smaller propylene molecules penetrate more easily than propane. However, as already mentioned, we can not absolutely rule out the opportunity of gas transmission via interlayer areas^104^.

With the addition of layer C (ZIF-8) and the formation of the fully nano-porous MFI/GO/ZIF-8 membrane (consisting of three layers A-C), a total C_3_H_6_ permeance of 50 GUP and a selectivity of 136 were achieved (see Fig. 1). The key factor in achieving high permeance was the synergistic collaboration of the three layers of the nanostructured membrane (layers A-C) to reduce the overall membrane thickness (3.65 µm) compared to the permeable layer D (thickness of 1.415 µm). It appears that this factor is also effective in increasing the selectivity of the MFI/GO/ZIF-8 membrane. The estimated selectivities of layers A-D are also presented in Table 3.

On the other hand, the simultaneous enhancement of both selectivity and permeability of the MFI/GO/ZIF-8 membrane is most likely due to the exchange of ZIF-8-like materials within the layers. The crystallization of the ZIF-8 matrix within the confined microscopic regions between the Graphene Oxide nanosheets/MFI nanoparticles with condensed packing may also lead to the formation of crystalline or partially amorphous regions with dimensions much smaller than the effective pore size of ZIF-8^56^.

After controlling the crystal size by software, according to Table 3, the A layer selectivity increased from 232.7 to 364, B layer selectivity increased from 1981.2 to 2358, C layer selectivity increased from 87 to 123, and D layer selectivity increased from 38 to 56.3. Additionally, the overall permeability of propylene increased in the MFI/GO/ZIF-8 membrane, and the overall membrane selectivity showed a growth of 23.1% (from 136 to 177) (see Table 2).

### Comparison of the maximum selectivity and permeability of propylene prepared MFI/GO/ZIF-8 membrane with other MMMs reported in the literature

In this paper, a membrane was fabricated by combining all nanoporous materials with excellent separation properties using a simple and reproducible method. Table 10 reports a comparison of the maximum selectivity and permeance of propylene in the MFI/GO/ZIF-8 membrane with hybrid membranes available in the literature for propylene separation. These results demonstrate the excellent selectivity and permeance of propylene achieved in this study.

**Table 10:** Comparison of the maximum selectivity and permeability of propylene using the MFI/GO/ZIF-8 membrane with other similar membranes was published in the literature.

Membranes	C_3_H_6_ permeance )GPU(	C_3_H_6_/C_3_H_8_ selectivity	Operational conditions	Ref
MFI/GO/ZIF-8	~ 60	177	25°C , 2 bar	Present work
ZIF-8/MGO	51.7	35.3	25°C , 1 bar	^105^
ZIF-8/6FDA-DAM	0.27	27.5	35°C , ~ 0.1 MPa	^106^
Zr-fum-fu MOF/6FDA-DAM	0.29 ~ 0.43	15 ~ 20	35°C , 212 kPa	^107^
ZIF-8/PIM-6FDA-OH	2.17	20	35 °C , 2 bar	^108^
ZIF-8/MFI	61 ~ 68	146 ± 14	25 °C , 1 bar	^109^
ZIF-8/MFI	66.4 ± 12.5	72 ± 29	25 °C , 1 bar	^20^
ZIF-8-ZNi	47 ± 2.53	48.15 ± 3.28	25 °C , 1 bar	^48^
ZIF-8-ZCL	44.2 ± 1.71	11.47 ± 2.43	25 °C , 1 bar	^48^
ZIF-8-Zac	64.44 ± 7.6	4.01 ± 1.40	25 °C , 1 bar	^48^

## Conclusion

The permeability and selectivity of propylene in the MFI/GO/ZIF-8 all-nanoporous hybrid membrane were investigated with C_3_H_6_/C_3_H_8_ = 90/10 mixture. For this purpose, the A layer (pure MFI) was controlled in two stages to achieve the minimum and optimal pore size for increasing selectivity and overall permeability of propylene. In the first stage, three parameters including crystallization time, NaOH concentration, and aging time of the initial suspension were controlled, and in the second stage, the effect of three parameters including hydrothermal time, hydrothermal temperature, and NH_4_F concentration on the optimal pore size was evaluated using the Box–Behnken method.

The Design-Expert software predicted the optimal crystal size values to achieve the mentioned goal in the first stage of synthesis, which were 177.8 nm, 12 h of aging time, 20 h of crystallization time, and 0.35 NaOH concentration, and in the second stage of synthesis, which were 120.53 nm of crystal size, 13 h of hydrothermal time, 180˚C of hydrothermal temperature, and 0.05 NH_4_F concentration. The pore size control results showed that the optimum pore size decreased from 177.8 nm to 120.53 nm.

Based on these results, the selectivity of propylene and the permeability of the MFI/GO/ZIF-8 membrane were further tested using the step-by-step membrane fabrication method. The permeability and overall selectivity of propylene in the mentioned membrane increased from 50 to 60 GPU and from 136 to 177, respectively, before and after controlling the crystal size in the zeolite layer. As a result, the prepared membrane exhibited a significant increase of 23.1% in selectivity and 16.7% in propylene permeability following the pore size control of crystal size. Additionally, in each of the A-D layers, the selectivity of propylene increased by 36%, 15.9%, 29.3%, and 32.5% respectively, by controlling the pore size in the first layer.

## Data Availability

All data generated or analyzed during this study are included in this published article.
